# The impact of hepatic steatosis on outcomes of colorectal cancer patients with liver metastases: A systematic review and meta-analysis

**DOI:** 10.3389/fmed.2022.938718

**Published:** 2022-09-08

**Authors:** Shengjie Yang, Renze Peng, Leiming Zhou

**Affiliations:** Department of Gastroenterology, Changxing County People’s Hospital, Huzhou, China

**Keywords:** colorectal cancer, liver metastasis, fatty liver, hepatic steatosis, mortality

## Abstract

**Background:**

It is unclear how hepatic steatosis impacts patient prognosis in the case of colorectal cancer with liver metastases (CRLM). The purpose of this review was to assess the effect of hepatic steatosis on patient survival and disease-free survival (DFS) in the case of CRLM.

**Methods:**

We examined the databases of PubMed, CENTRAL, Embase, Google Scholar, and ScienceDirect for studies reporting outcomes of CRLM patients with and without hepatic steatosis. We performed a random-effects meta-analysis using multivariable adjusted hazard ratios (HR).

**Results:**

Nine studies reporting data of a total of 14,197 patients were included. All patients had undergone surgical intervention. Pooled analysis of seven studies indicated that hepatic steatosis had no statistically significant impact on patient survival in CRLM (HR: 0.92 95% CI: 0.82, 1.04, I^2^ = 82%, *p* = 0.18). Specifically, we noted that there was a statistically significant improvement in cancer-specific survival amongst patients with hepatic steatosis (two studies; HR: 0.85 95% CI: 0.76, 0.95, I^2^ = 41%, *p* = 0.005) while there was no difference in overall survival (five studies; HR: 0.97 95% CI: 0.83, 1.13, I^2^ = 78%, *p* = 0.68). On meta-analysis of four studies, we noted that the presence of hepatic steatosis resulted in statistically significant reduced DFS in patients with CRLM (HR: 1.32 95% CI: 1.08, 1.62, I^2^ = 67%, *p* = 0.007).

**Conclusion:**

The presence of hepatic steatosis may not influence patient survival in CRLM. However, scarce data is suggestive of poor DFS in CRLM patients with hepatic steatosis. Further prospective studies taking into account different confounding variables are needed to better assess the effect of hepatic steatosis on outcomes of CRLM.

**Systematic review registration:**

[https://www.crd.york.ac.uk/prospero/#searchadvanced], identifier [CRD42022320665].

## Introduction

Colorectal cancer (CRC) is the second most common cancer in women and the third most common cancer amongst men worldwide (1). While the prevalence of this malignancy has been high in Western populations, recent data suggests that CRC is being increasingly diagnosed even in developing regions (2). The disease is known to be an age-associated malignancy with the median age of diagnosis being 67 years (3). Data from the United States reveals that of 140,000 patients diagnosed with CRC in 2018, approximately 60% of the patients were above the age of 65 years and these contributed to almost 70% of deaths during this period (4).

With improvements in tumor detection and therapeutic modalities, patient survival in CRC has improved over time (5). Nevertheless, colorectal liver metastases (CRLM) are still a major cause of mortality in these patients. Data suggests that more than half of the patients with CRC develop liver metastases during the illness and the median survival time is only 5–20 months in patients receiving no treatment for the metastatic disease (6, 7). Other than liver transplantation, surgical resection is the only curative treatment available for such patients which leads to a 5-year survival of up to 50% (8, 9). However, survival after hepatic resection depends on several factors like pre-existing hepatocellular damage, transfusion requirements, the size of the largest metastasis, and its distance to the resection margin (10). Recognition of such variables is important to characterize patients, develop a personalized management plan, and provide a realistic picture of survival and recurrence to CRLM patients.

One such important factor which has received limited attention in the prognosis of CRLM patients is hepatic steatosis. Also known as non-alcoholic fatty liver disease (NAFLD), hepatic steatosis is characterized by deposition of micro or macrovesicular lipid droplets in at least 5% of hepatocytes or more than 5% of liver weight without any signs of inflammation. While in most cases hepatic steatosis is the same as NAFLD, in some cases it may be attributable to alcohol as well (11). Irrespective of the etiology, in many patients, the disease gradually progresses to steatohepatitis and cirrhosis resulting in attenuated liver metabolic function and inflammation. Such deranged liver histology provides a fertile ground for seeding and colonization of CRC metastatic cells (12). To date, many studies have examined the effect of hepatic steatosis on outcomes of CRLM, however, with conflicting results. While some studies report no change in outcomes with hepatic steatosis (13, 14), others report poor patient survival and increased risk of recurrence (12, 15). Given the ambiguity in literature and the absence of a systematic review, the present study was designed to pool evidence on the effect of hepatic steatosis, irrespective of the etiology, on long-term outcomes of CRLM patients undergoing surgical intervention.

## Materials and methods

### Database search

The protocol of the review was registered on PROSPERO with registration no CRD42022320665. We looked into the databases of PubMed, CENTRAL, Embase, Google Scholar, and ScienceDirect for studies assessing the impact of hepatic steatosis on CRLM patients. This was done using the search terms “fatty liver,” “liver fat,” “hepatic steatosis,” “rectal cancer,” “colorectal cancer,” “colon cancer” and “metastasis.” The search strategy was common to all databases (Supplementary Table 1) and was performed by two of the study reviewers separately. The last date of the search was 28th March 2021. Once the initial search results were obtained, the results were exported and deduplicated. We then reviewed the unique results by reading the titles and abstracts to perform an initial screening. Only those studies which seemed to fulfill the eligibility criteria were downloaded for final screening. Inter-reviewer differences in the selection process were resolved in consultation with the third reviewer. We also undertook a hand-search of the reference list of the eligible studies to check for any missed relevant studies. The PRISMA guidelines were followed to report the review (16).

### Inclusion criteria

We included the following studies: (1) Cohort and case-control studies performed on individuals with CRLM undergoing surgical resection. (2) Studies comparing outcomes of patients with and without hepatic steatosis irrespective of the etiology. (3) Outcomes of interest were patient survival [overall survival (OS) and cancer-specific survival (CSS)] and disease-free survival (DFS). (4) Studies reporting multivariable-adjusted effect size with 95% confidence intervals (CI). (5) Follow-up duration of more than 6 months.

We excluded: (1) Studies reporting on a general cohort of CRC patients. (2) Studies on patients without any surgical intervention. (3) Studies reporting only crude outcomes. (4) Studies reporting only immediate mortality rates (in-hospital, 30-day, 90-day). (5) Studies only on hepatic fibrosis or steatohepatitis. (6) Non-English language studies. (7) Studies with duplicate data. In case studies had partial overlap of data, the study with the largest sample size was to be included.

### Data management and risk of bias

The following data was sourced from the studies: first author, publication year, type of the study, study location and database, inclusion criteria, diagnosis of hepatic steatosis, number of participants, demographic details, details of neoadjuvant chemotherapy, largest metastasis, nodal invasion outcomes, and follow-up.

Risk of bias was examined using the Newcastle-Ottawa scale (NOS) (17). The scale assesses the studies for selection of study population, comparability, and outcomes. The maximum points in the scale are nine. A study with ≤ 6, 7–8 and 9 points had high, moderate and low risk of bias respectively. Quality assessment was conducted by two reviewers with differences being resolved by the third reviewer.

### Statistical analysis

Outcome data reported as adjusted hazard ratios (HR) were pooled in the meta-analysis software “Review Manager” [RevMan, version 5.3; Nordic Cochrane Centre (Cochrane Collaboration), Copenhagen, Denmark; 2014] to obtain pooled HR with 95% CI in a random-effects meta-analysis model. The I^2^ scores were used to assess heterogeneity. I^2^ = 25–50% meant low, 50–75% meant medium, and more than 75% meant substantial heterogeneity. We also conducted a leave-one-out sensitivity analysis to assess the effect of each study on the overall results. Due to limited number of studies (less than 10), funnel plots were not used to judge for publication bias.

## Results

**FIGURE 1:**
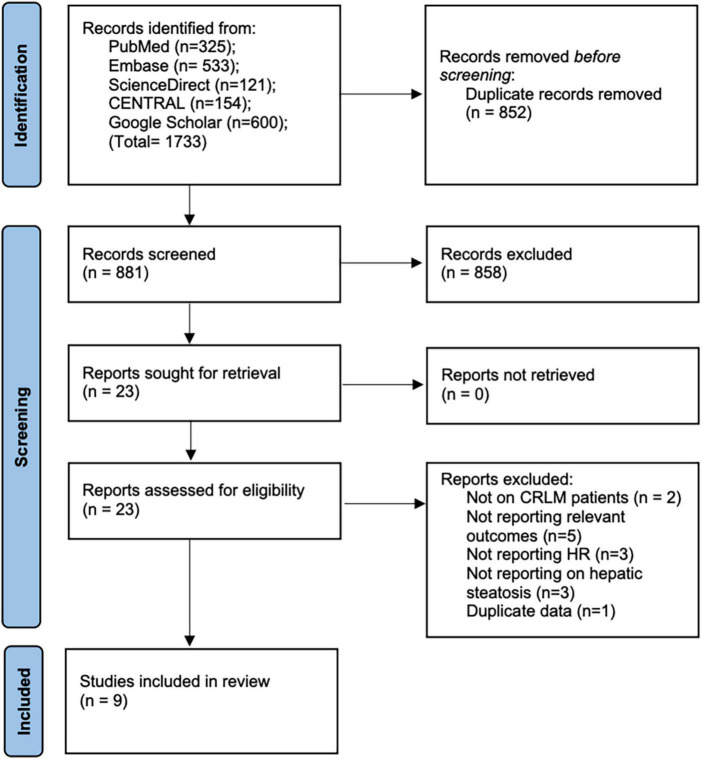
Study flow chart.

We retrieved 1,031 unique articles for review (Figure 1). After initial screening, 1,015 records were excluded. Of the 16 studies selected for full-text analysis, seven studies were excluded with reasons. A total of nine studies fulfilled the eligibility criteria and were included in this review (12–15, 18–22).

Baseline details of these studies are presented in Table 1. All were retrospective cohort studies published between 2013 and 2021. Two studies (14, 19) were multicentric reporting data from multiple countries across the world. One (12) was conducted on the Asian population while the rest were on Western populations. The study period was wide-ranging from 1987 to 2019. Hepatic steatosis was diagnosed histologically in all studies, except one (12) wherein radiological criteria were used. A total of 14,197 patients were included in all nine studies. The mean age was > 60 years in most studies. The proportion of male patients ranged from 32.2 to 70.1%. Three studies (20–22) did not report data on the use of neoadjuvant chemotherapy. Only one study (15) reported data on body mass index (BMI) in the study and control groups. For the remaining studies, the percentage of patients receiving neoadjuvant chemotherapy ranged from 0 to 100%. The incidence of nodal invasion in the study cohorts ranged from 30 to 71.8%. The studies had a follow-up ranging from a median of 7–127 months. The results of individual studies and the adjusted covariates in the analysis are presented in Table 2.

**TABLE 1:** Details of included studies.

Study	Location	Database	Period	Patient population	Diagnosis of fatty liver	Group	Sample size	Mean age	Male gender (%)	BMI (kg/m^2^)	NC (%)	Largest metastasis > 5 cm (%)	Lymph node invasion	Follow-up
van Dijk (15)	Netherlands	Maastricht University Medical Centre	2008–2013	CRLM patients who underwent partial hepatectomy	H	Fatty Liver Control	135 83	64.8 62.2	64 66	27.1 ± 3.7 25 ± 3.5	68 69	12 16	64 64	Median 56 months
Chen (12)	China	Second Affiliated Hospital of Zhejiang University School of Medicine	2012–2019	CRLM patients who underwent resection of the primary site and hepatectomy/RFA with curative intention	R	Fatty Liver Control	39 156	NR	66.7 69.2	NR	58.9 57.7	23.1 19.2	71.8 58.3	Median 7 months
Alabraba (22)	United Kingdom	Nottingham University Hospitals	2004–2017	CRLM patients who curative surgical resection	H	Fatty Liver Control	160 426	NR	NR	NR	NR	NR	NR	Median 23 months
Mahlmann (20)	Germany	Hannover Medical School	2000–2014	CRLM patients who underwent liver resection	H	Fatty Liver Control	277 46	NR	NR	NR	NR	NR	NR	NR
Ramos (21)	Spain	Hospital Universitario de Bellvitge	1990–2014	CRLM patients who underwent liver resection	H	Fatty Liver Control	421 513	62.6 62.9	70.1 67.8	NR	41.2 47	NR	NR	Median 47 months
Parkin (19)	Multicentric	LiverMetSurvey	1990–2011	CRLM patients who underwent liver resection with pre-operative chemotherapy	H	Fatty Liver Control	1675 1913	62.7 61.4	63.8 60.4	NR	100 100	23.3 23.9	66.2 66.4	Median 17.3 months
Viganò (13)	Switzerland	Mauriziano Umberto I Hospital of Torino	1998–2011	CRLM patients who underwent liver resection with pre-operative chemotherapy	H	Fatty Liver Control	73 250	NR	NR	NR	100 100	NR	NR	Median 36.9 months
Parkin (14)	Multicentric	LiverMetSurvey	1990–2011	CRLM patients who underwent liver resection without pre-operative chemotherapy	H	Fatty Liver Control	1793 3522	64.5 63.9	32.2 58.3	NR	0 0	30.8 59.7	30 59.9	Median 20 months
Hamady (18)	United Kingdom	Hampshire Hospitals	1987–2010	CRLM patients who underwent liver resection	H	Fatty Liver Control	927 1788	NR	68.9 61.3	NR	54.4 46.8	30.1 39.9	71.2 64.5	Median 34 months

OS, overall survival; DFS, disease free survival; CSS, cancer specific survival; CI, confidence interval; CRLM, colorectal liver metastases; NC, neoadjuvant chemotherapy; H, histological; R, radiological.

**TABLE 2:** Details of study results and covariates adjusted in the analysis.

Study	Outcomes [Hazard ratio (95% CI)]	Covariates adjusted
van Dijk (15)	OS: 1.8 (1, 3) DFS: 1.8 (1, 3)	Sex, age, American Society of Anesthesiologists, comorbidity, neoadjuvant chemotherapy, radical resection, Fong score
Chen (12)	DFS: 1.86 (1.23, 2.82)	Tumor location, number of liver metastasis, preoperative chemotherapy, surgery type, KRAS mutation, BRAF mutation
Alabraba (22)	DFS: 1.285 (1.086–1.520)	Synchronous/metachronous presentation, neoadjuvant chemotherapy, surgery type, tumor size, number of metastases, vascular invasion, perineural invasion, R0 resection margin
Mahlmann (20)	OS: 0.997 (0.987, 1.007)	NR
Ramos (21)	OS: 0.81 (0.63, 1.03)	Age, portal vein embolization, major liver resection, perioperative transfusion
Parkin (19)	CSS: 0.904 (0.796, 1.026)	Node positive primary, number of hepatic metastases > 3, carcinoembryonic antigen level > 60 ng/mL, tumor diameter ≥ 5 cm, positive resection margin, extra-hepatic disease
Viganò (13)	OS: 0.354 (0.183, 0.684)	Number of metastases, “N” stage, tumor diameter, extra-hepatic disease, adjuvant chemotherapy, chemotherapy related-liver injuries, tumor regression grade, percentage of viable cells on histology, positive resection margin
Parkin (14)	CSS: 0.806 (0.717, 0.905)	Node positive primary, number of hepatic metastases > 3, carcinoembryonic antigen level > 60 ng/mL, tumor diameter ≥ 5 cm, positive resection margin, extra-hepatic disease
Hamady (18)	OS: 1.08 (0.97, 1.21) DFS: 1.11 (1.01, 1.22)	Age, sex, lymph node status of the primary tumor, timing of liver metastases (synchronous versus metachronous), disease-free interval, presence of extrahepatic disease at time of diagnosis, largest hepatic metastasis diameter, number of liver metastases, lobar distribution (unilateral versus bilateral), carcinoembryonic antigen level, preoperative chemotherapy, resection margin status

NR, not reported.

### Meta-analysis

**FIGURE 2:**
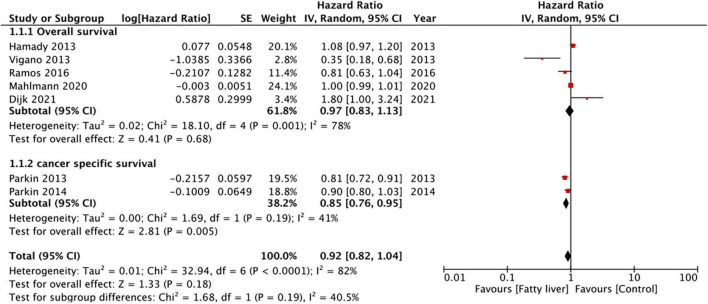
Meta-analysis of the impact of hepatic steatosis on patient survival in CRLM.

Five studies reported data on OS while two presented data on CSS. Pooled analysis of all seven studies indicated that hepatic steatosis had no statistically significant impact on patient survival in CRLM (HR: 0.92 95% CI: 0.82, 1.04, I^2^ = 82%, *p* = 0.18) (Figure 2). Individually, we noted that there was statistically significant improvement in CSS amongst patients with hepatic steatosis (HR: 0.85 95% CI: 0.76, 0.95, I^2^ = 41%, *p* = 0.005) while there was no difference in OS (HR: 0.97 95% CI: 0.83, 1.13, I^2^ = 78%, *p* = 0.68). On sensitivity analysis, there was no change in the significance of the results of patient survival on the exclusion of any study (Table 3).

**TABLE 3:** Results of sensitivity analysis.

Excluded study	Hazard ratio
**Patient survival**	
Hamady (18)	0.88 95% CI: 0.76, 1.03 I^2^ = 84% *p* = 0.11
Viganò (13)	0.95 95% CI: 0.86, 1.05 I^2^ = 79% *p* = 0.31
Ramos (21)	0.94 95% CI: 0.83, 1.06 I^2^ = 84% *p* = 0.33
Mahlmann (20)	0.89 95% CI: 0.74, 1.07 I^2^ = 82% *p* = 0.23
van Dijk (15)	0.91 95% CI: 0.81, 1.01 I^2^ = 83% *p* = 0.09
Parkin (14)	0.96 95% CI: 0.85, 1.08 I^2^ = 75% *p* = 0.49
Parkin (19)	0.93 95% CI: 0.80, 1.07 I^2^ = 84% *p* = 0.28
**Disease free survival**	
Hamady (18)	1.50 95% CI: 1.15, 1.96 I^2^ = 42% *p* = 0.003
Alabraba (22)	1.46 95% CI: 0.97, 2.20 I^2^ = 75% *p* = 0.07
Chen (12)	1.22 95% CI: 1.03, 1.43 I^2^ = 55% *p* = 0.02
van Dijk (15)	1.28 95% CI: 1.04, 1.57 I^2^ = 72% *p* = 0.02

CI, confidence interval.

**FIGURE 3:**
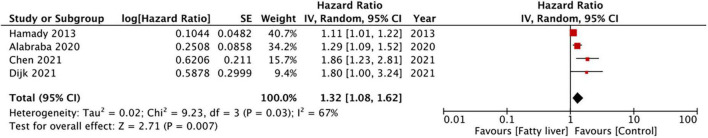
Meta-analysis of the impact of hepatic steatosis on DFS in CRLM.

Four studies reported data on DFS. On meta-analysis, we noted that the presence of hepatic steatosis resulted in statistically significant reduced DFS in patients with CRLM (HR: 1.32 95% CI: 1.08, 1.62, I^2^ = 67%, *p* = 0.007) (Figure 3). On the sequential exclusion of one study at a time, the results were still statistically significant with the HR ranging from 1.28 to 1.50 (Table 3). However, on the exclusion of the study of Alabraba (22), the results demonstrated a tendency of reduced DFS with hepatic steatosis but with non-significant results. Risk of bias analysis is presented in Table 4. The NOS score of the studies was either 7 or 8 indicating moderate quality.

**TABLE 4:** Risk of bias evaluation of individual studies.

Study	Selection				Comparability		Outcome			Total
	
	Representative of the exposed cohort	Selection of external cohort	Ascertainment of exposure	Outcome of interest does not present at start	Main factor	Additional factor	Assessment of outcome	Sufficient follow up	Adequacy of follow up	(9/9)
van Dijk (15)	+	+	+	+	+	+	+	+	0	8
Chen (12)	+	+	+	+	+	+	+	0	0	7
Alabraba (22)	+	+	+	+	+	+	+	+	0	8
Mahlmann (20)	+	+	+	+	+	+	+	0	0	7
Ramos (21)	+	+	+	+	+	+	+	+	0	8
Parkin (19)	+	+	+	+	+	+	+	+	0	8
Viganò (13)	+	+	+	+	+	+	+	+	0	8
Parkin (14)	+	+	+	+	+	+	+	+	0	8
Hamady (18)	+	+	+	+	+	+	+	+	0	8

## Discussion

NAFLD is one of the most common causes of chronic liver disease worldwide. NAFLD is characterized by the accumulation of excess fat in the liver that is not attributable to alcohol. Indeed, the increased prevalence of metabolic syndrome and obesity has expanded the presence of NAFLD (11). Global estimates indicate that around 25.24% of the world population suffers from the disease (23) with its spectrum including hepatic steatosis, hepatic steatohepatitis, and hepatic fibrosis/cirrhosis in advanced cases (24). Due to the complex nature of the disease, the relationship between NAFLD and malignancies has been quite intriguing. In a recent meta-analysis, Liu et al. (25) have suggested that NAFLD is associated with a statistically significant increased risk of CRC, intrahepatic cholangiocarcinoma, extrahepatic cholangiocarcinoma, breast, gastric, pancreatic, prostate, and esophageal cancer. While the increased risk of CRC is postulated by this review, it is unclear if the disease impacts outcomes of CRC especially those with liver metastases.

The focus of our meta-analysis was to assess if the presence of hepatic steatosis, irrespective of its etiology, leads to poor patient survival and increased risk of recurrence in patients with CRLM. We specifically chose to assess this risk only with hepatic steatosis as it is the most common phenotype of NAFLD and only 10–30% of patients develop steatohepatitis and fibrosis (11). Commensurating with the prevalence, the majority of the studies in literature also reported data on hepatic steatosis rather than steatohepatitis or fibrosis. For the first meta-analysis, we noted that the presence of hepatic steatosis did not affect patient survival. On examination of individual study data, one can note that the study of Viganò (13) and Parkin (14) reported a protective role of hepatic steatosis on patient survival while the others noted no effect on mortality rates. The contrasting result is difficult to explain because hepatic steatosis is a pathological condition that is associated with an increased risk of cancer and even CRLM recurrence (according to our results). One plausible reason could be the association of hepatic steatosis with obesity. Pathak et al. (26) have shown that BMI but not diabetes independently predicts the development of hepatic steatosis. Furthermore, there is evidence to suggest the occurrence of the “obesity paradox” wherein high BMI is associated with better survival in CRC patients (27). Considering the complex association between NAFLD, BMI, body composition, and patient survival it is possible that the improved survival with hepatic steatosis in these studies could have been due to the excessive influence of one factor over the other. The lack of such contrasting results in the remaining studies along with the stability of the outcome on sensitivity analysis lends support to the conclusion that hepatic steatosis may not influence patient survival in CRLM. Our results concur with other studies which could not be included in our meta-analysis. Pathak et al. (26) in their study of 102 patients undergoing hepatectomy for CRLM has shown no difference in mean survival in patients with and without hepatic steatosis (28.6 vs. 32.3 months). Zhao et al. (28) have also noted no impact of steatohepatitis on patient survival after CRLM. While our review was focused only on the long-term impact of hepatic steatosis, studies assessing short-term morbidity and mortality outcomes have produced conflicting results as well. Older studies (29, 30) have shown that the presence of hepatic steatosis increases the risk of early morbidity and mortality in patients undergoing liver resections, however, more recent data segregating steatosis from steatohepatitis shows no such effect (31, 32).

In the second meta-analysis of just four studies, we noted that the presence of hepatic steatosis significantly reduced DFS in CRLM patients. Individually, the results were consistent across the four included studies but the lower end of 95% CI was close to 1 in the majority of the studies (indicative of no difference). Thus, the results should be interpreted with caution and there is a need for further data to strengthen the credibility of the current evidence. However, in concurrence with our results, studies not included in the meta-analysis have also shown a similar impact of hepatic steatosis on DFS in CRLM. Molla et al. (33) in an analysis of 60 patients undergoing curative resection for CRLM noted an increased risk of recurrence in patients with NAFLD. Expanding further from hepatic steatosis to a more severe form of NAFLD, Kondo et al. (34) in a cohort of 953 CRC patients have shown that hepatic fibrosis significantly increases the risk of hepatic-specific recurrence. Similarly, Narayan et al. (35) assessing the influence of hepatic parenchymal disease (i.e., steatosis, hepatocyte ballooning, lobular inflammation, and fibrosis) on CRLM outcomes have also noted reduced DFS with liver disease.

The mechanism behind increased recurrence in CRLM has been attributed to the favorable microenvironment created by hepatic steatosis for tumor seeding. The pathophysiology of hepatic steatosis is modulated by several cytokines like transforming growth factor β, interleukins, and tumor necrosis factor-α which in turn also promote the development of liver metastases in CRC patients (36, 37). Dysregulated presence of such cytokines causes a dual autocrine and paracrine effect resulting in an inflammatory milieu that creates a pre-metastatic niche for CRLM (38). Animal studies have also reported that the presence of steatosis significantly increases the risk of liver metastases (39). The suggested mechanism is that fatty acid transporter protein 1 transports lipolytic products into cancer cells and promotes tumor growth by mitochondrial oxidation (39). The presence of hepatic steatosis also leads to extracellular matrix remodeling and reorganization thereby creating a fibrotic niche for CRLM (40).

An important factor that can influence the risk of both steatosis and prognosis of patients with CRLM is neoadjuvant chemotherapy. Preoperative chemotherapy can improve disease control and convert initially unresectable malignancies for surgery in selected patients. In the case of resectable disease, the therapy can lower the burden of metastases and improve surgical outcomes (13). However, chemotherapy itself is associated with liver injuries ranging from sinusoidal obstruction syndrome lesion, nodular regenerative hyperplasia, steatosis, and steatohepatitis which in turn can potentially worsen surgical morbidity and mortality (20). However, liver injuries with chemotherapy are regimen-specific. Oxaliplatin-based regimens and irinotecan-based regimens are known to increase the risk of sinusoidal injury and steatohepatitis respectively, on the other hand, bevacizumab with FOLFOX reduces the risk of grade 2 or greater sinusoidal injury (41). Amongst the included studies, there was a large variation in the percentage of patients receiving neoadjuvant chemotherapy (0–100%) while two studies did not report data on the same. We were unable to differentiate between chemotherapy-induced steatosis and steatosis present before the development of CRLM. The wide variation and a limited number of studies also precluded a subgroup analysis based on preoperative chemotherapy.

Other limitations of our review need to be specified as well. Foremost, the limited number of studies and the retrospective nature of data is a significant drawback. Retrospective studies are inherently prone to selection bias. Furthermore, despite including nine studies in the review, not all studies reported data on patient survival and DFS which further decreased the statistical power of each analysis. Secondly, baseline details of study participants were either not available or varied across the included studies. Differences in important variables like tumor location, tumor size, nodal invasion, number and size of metastases, adjuvant therapies, etc., could have skewed the study results. An attempt was made to skirt this limitation by using only multivariable-adjusted data, however, the difference between the studies in the adjusted outcomes could not be negated. Thirdly, our meta-analysis could not differentiate between the different grades of hepatic steatosis due to a lack of adequate data from the included studies. Patients were grouped based on just presence and absence of hepatic steatosis. Also due to limited data on steatohepatitis and hepatic fibrosis in literature, our review only focused on hepatic steatosis. Lastly, hepatic steatosis could be either due to NAFLD or in some cases may be attributable to alcohol. The data of the included studies precluded any differentiation in outcomes based on the drivers of steatosis.

Our findings have clinical implications. Since initial limited data is indicative of poor DFS in patients with hepatic steatosis, we believe clinicians should include this point in patient-doctor interactions and aggressively monitor hepatic steatosis patients for recurrence. However, considering the scarce data the findings need to be concurred by additional studies. Also, future prospective studies with a large sample size are needed to better elucidate the impact of the entire spectrum of NAFLD on the prognosis of CRLM patients.

## Conclusion

Our review which is the first to assess the impact of hepatic steatosis on outcomes of CRLM patients indicates that the presence of hepatic steatosis may not influence patient survival. However, scarce data is suggestive of poor DFS in CRLM patients with hepatic steatosis. Further prospective studies taking into account different confounding variables are needed to better assess the effect of hepatic steatosis on outcomes of CRLM.

## Data availability statement

The original contributions presented in this study are included in the article/Supplementary material, further inquiries can be directed to the corresponding author/s.

## Author contributions

SY and RP conceived and designed the study. SY, RP, and LZ collected the data and performed the analysis. LZ was involved in the writing of the manuscript and responsible for the integrity of the study. All authors have read and approved the final manuscript.
