# Supplementary material for: The impact of hepatic steatosis on outcomes of colorectal cancer patients with liver metastases: A systematic review and meta-analysis
Source: Front Med (Lausanne). 2022 Sep 8;9:938718. doi: 10.3389/fmed.2022.938718 (PMC9498207; doi:10.3389/fmed.2022.938718)
Supplement: Supplementary file 1 [file Table_1.docx]

Supplementary Table 1: Search strategy

| **Query** | **Search Details** |
| --- | --- |
| ((((fatty liver) OR (liver fat)) OR (hepatic steatosis)) AND (rectal cancer)) AND (metastases) | ("fatty liver"[MeSH Terms] OR ("fatty"[All Fields] AND "liver"[All Fields]) OR "fatty liver"[All Fields] OR (("liver"[MeSH Terms] OR "liver"[All Fields] OR "livers"[All Fields] OR "liver s"[All Fields]) AND "fat"[All Fields]) OR ("fatty liver"[MeSH Terms] OR ("fatty"[All Fields] AND "liver"[All Fields]) OR "fatty liver"[All Fields] OR ("hepatic"[All Fields] AND "steatosis"[All Fields]) OR "hepatic steatosis"[All Fields])) AND ("rectal neoplasms"[MeSH Terms] OR ("rectal"[All Fields] AND "neoplasms"[All Fields]) OR "rectal neoplasms"[All Fields] OR ("rectal"[All Fields] AND "cancer"[All Fields]) OR "rectal cancer"[All Fields]) AND ("metastasation"[All Fields] OR "metastasic"[All Fields] OR "metastasing"[All Fields] OR "metastasise"[All Fields] OR "metastasised"[All Fields] OR "metastasises"[All Fields] OR "metastasising"[All Fields] OR "metastasization"[All Fields] OR "metastasizes"[All Fields] OR "metastasizing"[All Fields] OR "neoplasm metastasis"[MeSH Terms] OR ("neoplasm"[All Fields] AND "metastasis"[All Fields]) OR "neoplasm metastasis"[All Fields] OR "metastase"[All Fields] OR "metastases"[All Fields] OR "metastasize"[All Fields] OR "metastasized"[All Fields]) |
| ((((fatty liver) OR (liver fat)) OR (hepatic steatosis)) AND (colon cancer)) AND (metastases) | ("fatty liver"[MeSH Terms] OR ("fatty"[All Fields] AND "liver"[All Fields]) OR "fatty liver"[All Fields] OR (("liver"[MeSH Terms] OR "liver"[All Fields] OR "livers"[All Fields] OR "liver s"[All Fields]) AND "fat"[All Fields]) OR ("fatty liver"[MeSH Terms] OR ("fatty"[All Fields] AND "liver"[All Fields]) OR "fatty liver"[All Fields] OR ("hepatic"[All Fields] AND "steatosis"[All Fields]) OR "hepatic steatosis"[All Fields])) AND ("colonic neoplasms"[MeSH Terms] OR ("colonic"[All Fields] AND "neoplasms"[All Fields]) OR "colonic neoplasms"[All Fields] OR ("colon"[All Fields] AND "cancer"[All Fields]) OR "colon cancer"[All Fields]) AND ("metastasation"[All Fields] OR "metastasic"[All Fields] OR "metastasing"[All Fields] OR "metastasise"[All Fields] OR "metastasised"[All Fields] OR "metastasises"[All Fields] OR "metastasising"[All Fields] OR "metastasization"[All Fields] OR "metastasizes"[All Fields] OR "metastasizing"[All Fields] OR "neoplasm metastasis"[MeSH Terms] OR ("neoplasm"[All Fields] AND "metastasis"[All Fields]) OR "neoplasm metastasis"[All Fields] OR "metastase"[All Fields] OR "metastases"[All Fields] OR "metastasize"[All Fields] OR "metastasized"[All Fields]) |
| ((((fatty liver) OR (liver fat)) OR (hepatic steatosis)) AND (colorectal cancer)) AND (metastases) | ("fatty liver"[MeSH Terms] OR ("fatty"[All Fields] AND "liver"[All Fields]) OR "fatty liver"[All Fields] OR (("liver"[MeSH Terms] OR "liver"[All Fields] OR "livers"[All Fields] OR "liver s"[All Fields]) AND "fat"[All Fields]) OR ("fatty liver"[MeSH Terms] OR ("fatty"[All Fields] AND "liver"[All Fields]) OR "fatty liver"[All Fields] OR ("hepatic"[All Fields] AND "steatosis"[All Fields]) OR "hepatic steatosis"[All Fields])) AND ("colorectal neoplasms"[MeSH Terms] OR ("colorectal"[All Fields] AND "neoplasms"[All Fields]) OR "colorectal neoplasms"[All Fields] OR ("colorectal"[All Fields] AND "cancer"[All Fields]) OR "colorectal cancer"[All Fields]) AND ("metastasation"[All Fields] OR "metastasic"[All Fields] OR "metastasing"[All Fields] OR "metastasise"[All Fields] OR "metastasised"[All Fields] OR "metastasises"[All Fields] OR "metastasising"[All Fields] OR "metastasization"[All Fields] OR "metastasizes"[All Fields] OR "metastasizing"[All Fields] OR "neoplasm metastasis"[MeSH Terms] OR ("neoplasm"[All Fields] AND "metastasis"[All Fields]) OR "neoplasm metastasis"[All Fields] OR "metastase"[All Fields] OR "metastases"[All Fields] OR "metastasize"[All Fields] OR "metastasized"[All Fields]) |
